# Psychological Resilience as a Protective Factor for Depression and Anxiety Among the Public During the Outbreak of COVID-19

**DOI:** 10.3389/fpsyg.2020.618509

**Published:** 2021-01-22

**Authors:** Shasha Song, Xin Yang, Hua Yang, Ping Zhou, Hui Ma, Changjun Teng, Haocheng Chen, Hongxia Ou, Jijun Li, Carol A. Mathews, Sara Nutley, Na Liu, Xiangyang Zhang, Ning Zhang

**Affiliations:** ^1^Nanjing Brain Hospital Affiliated to Nanjing Medical University, Nanjing, China; ^2^Department of Medical Psychology, The Affiliated Brain Hospital of Nanjing Medical University, Nanjing, China; ^3^Department of Psychiatry, University of Florida, Gainesville, FL, United States; ^4^Department of Epidemiology, University of Florida, Gainesville, FL, United States; ^5^CAS Key Laboratory of Mental Health, Institute of Psychology, Chinese Academy of Sciences, Beijing, China

**Keywords:** COVID-19, depression, anxiety, related factors, public

## Abstract

**Background:**

Psychological resilience may reduce the impact of psychological distress to some extent. We aimed to investigate the mental health status of the public during the outbreak of coronavirus disease 2019 (COVID-19) and explore the level and related factors of anxiety and depression.

**Methods:**

From February 8 to March 9, 2020, 3,180 public completed the Zung’s Self-Rating Anxiety Scale (SAS) for anxiety, Zung’s Self-Rating Depression Scale (SDS) for depression, the Connor–Davidson resilience scale (CD-RISC) for psychological resilience, and the Simplified Coping Style Questionnaire (SCSQ) for the attitudes and coping styles.

**Results:**

The number of people with depressive symptoms (SDS > 53) was 1,303 (the rate was 41.0%). The number of people with anxiety symptoms (SAS > 50) was 1,184 (the rate was 37.2%). The depressed group and anxiety group had less education, more unmarried and younger age, as well as had significant different in SDS total score (*P* < 0.001), SAS total score (*P* < 0.001), CD-RISC total score (*P* < 0.001), and SCSQ score (*P* < 0.001). The binary logistic regression showed that female (*B* = -0.261, *P* = 0.026), strength (*B* = -0.079, *P* = 0.000), and the subscales of active coping style in SCSQ (*B* = -0.983, *P* = 0.000) remained protective factors and passive coping style (*B* = 0.293, *P* = 0.003) and higher SAS score (*B* = 0.175, *P* = 0.000) were risk factors for depression. Optimism (*B* = -0.041, *P* = 0.015) in CD-RISC was a protective factor, and passive coping styles (*B* = 0.483, *P* = 0.000) and higher SDS score (*B* = 0.134, *P* = 0.000) were risk factors for anxiety.

**Limitations:**

This study adopted a cross-sectional design and used self-report questionnaires.

**Conclusion:**

The mental health of the public, especially females, the younger and less educational populations, and unmarried individuals, should be given more attention. Individuals with high level of mental resilience and active coping styles would have lower levels of anxiety and depression during the outbreak of COVID-19.

## Introduction

Since the start of the global pandemic caused by the novel coronavirus SARS-CoV-2 late in 2019 (COVID-19) pandemic, the total number of cases worldwide has already exceeded the number of confirmed cases in China (Liu J. J. et al., 2020). Although initially severely affected by the outbreak, China has since made significant progress in the prevention and control of the infection that causes COVID-19. To date, the country has returned to daily life, and production and traffic have resumed in an orderly manner. However, the coronavirus pandemic continues to escalate throughout the world. The control of the epidemic and efforts to prevent further spread in China has transitioned from anti-proliferation of the virus locally to anti-import of the virus from outside of China’s borders (Ding et al., 2020), in addition to ongoing efforts to continue to prevent a rebound of infections domestically.

An increasing number of countries have indicated heightened public anxiety about being infected, and China is no exception (Bao et al., 2020). A recent survey on the psychological status of the population during the early stage of the epidemic by Qiu et al. (2020) showed that, among 52,730 individuals surveyed via questionnaire in mainland China, nearly 35% of the respondents reported experiencing psychological distress. Patients, health professionals, and the public are under insurmountable psychological pressure, which increases their risk for various psychological problems such as anxiety, fear, depression, and insomnia (Li W. et al., 2020). Surveys have shown us the pressures faced by medical staff, such as their responsibility to care for infected patients and their close contact with their families, sometimes in the face of public inquiries (Li W. et al., 2020). The public may be less psychologically prepared than medical workers and more fearful of the consequences of infection with a potentially lethal new virus. In addition, the persistent stress of the current situation has made people respond unpredictably and uncontrollably, while those in isolation may experience boredom, loneliness, and anger.

A meta-analysis examining the psychological state of individuals during the pandemic of COVID-19 in China showed increases in rates of anxiety and depression to 44.5 and 18.9%, respectively, and the rate of individuals experiencing negative psychological symptoms of comprehensive psychological symptoms was 72.9% (Wei et al., 2020). However, the psychological factors related to the development (or prevention) of symptoms such as anxiety and depression were not explored.

Given the ongoing nature of the COVID-19 pandemic and the profound and widespread effects on mental health worldwide, there is a need to identify factors (such as psychological resilience) that may protect against the development of anxiety, depression, and other psychological problems. Resilience is the psychological trait of having positive dispositions that enable individuals to effectively cope with stressful situations (Ehrich et al., 2017). Studies suggest both that the existence of psychological resilience is universal and that resilience has protective effects on the physical and mental status of individuals experiencing or facing adversity (Lee et al., 2018).

The most common way to assess psychological resilience is through self-report measures such as the Connor-Davidson Resilience Scale (CD-RISC; Sidheek et al., 2017). The CD-RISC assess three dimensions commonly associated with psychological resilience: tenacity, strength, and optimism. The tenacity dimension describes an individual’s equanimity, promptness, perseverance, and sense of control when facing situations of hardship and challenge. The strength dimension reflects an individual’s ability to recover from setbacks, including their propensity to become more (rather than less) energetic after experiencing setbacks. The optimism dimension measures an individual’s perception of the positive aspects of situations. Individuals with higher scores on the optimism dimension show an enhanced ability to recover after experiencing ups and downs in their daily life relative to those who have lower scores on this dimension (Yu and Zhang, 2007).

The present study aimed to explore the impact and dynamic changes of the mental health of the public in China during the ongoing COVID-19 pandemic, and in particular, to explore the levels of anxiety, depression and related psychological factors, and their relationships to psychological resilience and coping styles. We predicted that high levels of psychological resilience would be associated with lower levels of anxiety and depression and increased abilities to cope with the ongoing stresses of daily life during the pandemic. If substantiated, the findings resulting from this study would provide a theoretical basis and suggest possible viable strategies for psychological interventions during COVID-19 (Li Z. et al., 2020).

## Materials and Methods

All data were collected by Department of Medical Psychology of the affiliated Brain Hospital of Nanjing Medical University. All participants signed informed consent documents, and all procedures were approved by the Institutional Review Board of the affiliated Nanjing Brain Hospital of Nanjing Medical University. Questionnaires were organized by two psychiatrists and psychologists and delivered online, then updated day by day, and after a month the questionnaires were collected and analyzed according to the conditions as follows.

### Design and Procedures

The self-report questionnaire used in this study was designed to survey levels of anxiety, depression, psychological resilience, and coping styles in addition to basic demographic information (age, sex, marital status, and education level). Questionnaires were delivered to the public online via WenJuanXing software and the WeChat app, and the online official account of Nanjing Brain Hospital between February 8 to March 9, 2020, to avoid the risk of face-to-face infection during the peak period of the COVID-19 epidemic in mainland China.

### Subjects

Participants included members of the public in China who did not have a current or ever diagnosis of COVID-19. Potential participants were excluded from the study if they had: (1) a history of severe mental disorders which affect brain metabolism such as diabetes or thyroid disease, etc. (2) who had encountered a significant life event in the past 6 months, such as losing relatives, experiencing trauma, etc. (3) Those such as prevention and control frontline personnel including medical staff and their family members, diagnosed or suspected COVID-19 patient. Of the 3,960 questionnaires that were distributed, 3,180 were considered valid and were included in this study, while 780 were considered invalid and were excluded, for a validity rate of 80.30%. Questionnaires were considered to be invalid if they were not public. Questionnaires were considered to be invalid if they were completed in a very rapid time frame, had a very high repetition rate of responses, or were missing data for critical questions or sections.

### Measurements of Psychological Distress

#### Depression and Anxiety

The Self-rating Depression Scale (SDS; Zhengyu and Yufen, 1984a) and Self-rating Anxiety Scale (SAS; Zhengyu and Yufen, 1984b) were used to assess levels of anxiety and depression. The depression scale is based on Zung’s SDS, developed by W.K. Zung in 1965. The anxiety scale is based on Zung’s SAS, developed by W.K. Zung in 1971. Both scales were translated into their Chinese versions, with a high reliability coefficient for different populations in China. Each scale includes 20 items each scored on a four point Likert scale that assesses frequency or severity of symptoms of either depression or anxiety. “1” means no or little time, “2” represents a small amount of time, “3” represents a lot of time, and “4” represents most or all of the time. Reverse scoring questions are rated “4, 3, 2, and 1.” Self-assessment scale evaluation method: first explain the evaluation method, meaning and requirements to the pants participants, and the participants will fill in it according to the actual situation. Higher total scores indicate more severe depression or anxiety.

#### Psychological Resilience

Psychological resilience was measured using the CD-RISC (Connor and Davidson, 2003), translated from English into Chinese. The CD-RISC contains 25 items, each scored on a 5-point Likert scale and assesses three factors—Tenacity, Strength, and Optimism, The reliability coefficient of the Chinese version of CD-RISC is 0.91 (Yu and Zhang, 2007).

#### Coping Styles

Coping style was measured using the Simplified Coping Style Questionnaire (SCSQ). The scale was compiled by Xie Yaning using both domestic and foreign cognition theories about coping styles, combined with the characteristics of the Chinese population, The scale has excellent reliability 0.90 (Ya-ning, 1998). The SCSQ assesses both attitudes and coping styles of participants regarding specific life events or difficulties encountered in their daily lives. The scale consists of 20 items, each scored from 0 to 3 and divided into two dimensions: the positive response dimension is comprised of 12 items, and the negative response dimension is comprised of 8 items (Duanwei and Jingxuan, 2014). The higher the score is, the more habitually the coping style used.

### Statistical Methods

The data were organized and analyzed using SPSS 22.0 software. Quantitative measures of anxiety and depression were converted into categorical depression/anxiety groups using cutoff scores. Depression and anxiety groups were not mutually exclusive. Individuals who scored above 53 on the SDS questionnaire were considered to be in the depressed group, while those who scored below were considered to be in the non-depressed group (Quan-quan and Li, 2012). Individuals who scored above 50 on the SAS questionnaire were considered to be in the anxiety group, while those who scored below were considered to be in the non-anxiety group (Xiaoyang, 2011).

We first compared the demographic and correlation variables between the depressed and non-depressed groups and between the anxious and non-anxious groups using ANOVA for continuous variables, and chi-square test for categorical variables. Binary logistic regression was used to jointly analyze the factors that potentially influences depression and anxiety *P* < 0.05 was considered statistically significant. Pearson or Spearman correlations were used to explore associations between SDS, SAS score and demographic or assessments. Bonferroni correction was performed to adjust for multiple tests (α = 0.05/9 = 0.006). Quantitative data are reported as means ± standard deviation (*x* ± *s*) and categorical data as numbers and percents (n, %).

## Results

### Demographic Features

The survey comprised 3,180 individuals—886 men (27.9%) and 2,294 women (72.1%) and 886 men (27.9%). The whole sample average age was 34.09 ± 12.48 years, the education levels were the following: 824 (25.9%) with less than 12 years of education, 1,967 cases (61.9%) with 12 to 16 years of education, and 389 cases (12.3%) with more than 16 years of education. The marital statuses were as follows: 1,067 unmarried cases (33.6%), 1,953 married cases (61.4%), and 160 other cases (divorced/widowed; 5.0%).

From the point of view depression group, the average age was 29.16 ± 13.63 years. The education levels were the following: 401 (30.8%) participants had less than 12 years of education, 772 had (59.2%) 12 to 16 years of education, and 130 (10.0%) had more than 16 years of education. Forty four percent (*n* = 573) of participants were unmarried, 51% (*n* = 665) were married (51.0%), and 5% (*n* = 65) were divorced or widowed. The mean depression score for the overall sample was 52.89 ± 15.21, with 41% (*n* = 1,303) meeting cutoff criteria for depression (total score > 53).

From the point of view anxiety group, the average age was 30.91 ± 13.56 years. The education levels were the following: 346 (29.2%) participants had less than 12 years of education, 709 had (59.9%) 12 to 16 years of education, and 129 (10.9%) had more than 16 years of education. 39.2% (*n* = 464) of participants were unmarried, 55.3% (*n* = 655) were married (51.0%), and 5.5% (*n* = 65) were divorced or widowed. The mean anxiety score for the overall sample was 48.77 ± 11.45, with 37.2% (*n* = 1,184) meeting cutoff criteria for anxiety (total score > 50).

Demographic and psychological characteristics of the depressed and non-depressed groups are shown in Table 1. The depressed group was significantly more likely to be female, younger, and unmarried, and had lower educational attainment than the non-depressed group (Table 1). The same patterns were seen for the anxious and non-anxious groups, although there were no differences in the proportion of women in the anxious and non-anxious groups (Table 2).

**TABLE 1:** Social demographics and psychological assessments of people with depression and non-depression.

	**Depressed group (*n* = 1,303)**		**Non-depressed group (*n* = 1,877)**			
	***n***	**%**	***n***	**%**	***F***	***p***
**Gender**					6.649	0.01
Male	331	25.4	555	29.6		
Female	972	74.6	1,322	70.4		
**Age**					64.923	<0.001
<18	251	19.3	188	10.0		
18–55	1,010	77.5	1,573	83.8		
>55	42	3.2	116	6.2		
**Education**					31.125	<0.001
<12 years	401	30.8	423	22.5		
12–16 years	772	59.2	1,195	63.7		
>16 years	130	10.0	259	13.8		
**Marital status**					81.377	<0.001
Unmarried	573	44.0	494	26.3		
Married	665	51.0	1,288	68.6		
Others	65	5.0	95	5.1		

	**Mean**	**SD**	**Mean**	**SD**	**F/t**	***p***

Age	29.16	13.63	37.52	10.31	64.923	<0.001
SDS total score	67.95	11.17	42.43	6.21	82.341	<0.001
SAS total score	57.22	11.41	42.91	6.92	43.940	<0.001
**CD-RISC Total score**	54.12	19.27	72.82	15.01	−30.648	<0.001
Tenacity	26.74	10.37	36.10	8.57	−27.763	<0.001
Strength	19.12	6.61	25.48	5.03	−30.820	<0.001
Optimism	8.26	3.53	11.24	2.97	−25.727	<0.001
**SCSQ**						
Active coping	1.75	0.60	2.26	0.45	−27.426	<0.001
Passive coping	1.42	0.60	1.34	0.56	3.806	<0.001

*SAS, Self-rating Anxiety Scale; SDS, Self-rating Depression Scale; CD-RISC, Connor-Davidson Resilience Scale; and SCSQ, Simplified Coping Style Questionnaire.*

**TABLE 2:** Social demographics and psychological assessments of people with anxiety and non-anxiety.

	**Anxious group (*n* = 1,184)**		**Non-anxious group (*n* = 1,996)**			
	***n***	**%**	***n***	**%**	***F***	***p***
**Gender**					0.056	0.814
Male	327	27.6	559	28.0		
Female	857	72.4	1,437	72.0		
**Age**					9.920	0.005
<18	185	15.6	254	12.7		
18–55	955	80.7	1,628	81.6		
>55	44	3.7	114	5.7		
**Education**					11.270	0.003
<12 years	346	29.2	478	23.9		
12–16 years	709	59.9	1,258	63.0		
>16 years	129	10.9	260	13.0		
**Marital status**					16.682	<0.001
Unmarried	464	39.2	603	30.2		
Married	655	55.3	1,298	65.0		
Others	65	5.5	95	4.8		

	**Mean**	**SD**	**Mean**	**SD**	**F/t**	***p***

Age	30.91	13.56	35.98	11.38	9.920	<0.001
SDS total score	65.78	14.32	45.23	9.48	48.623	<0.001
SAS total score	60.40	9.51	41.88	5.24	70.738	<0.001
**CD-RISC Total score**	55.20	19.81	71.07	16.26	−24.501	<0.001
Tenacity	27.29	10.69	35.21	9.04	−22.294	<0.001
Strength	19.50	6.74	24.88	5.49	−24.534	<0.001
Optimism	8.41	3.58	10.98	3.13	−21.176	<0.001
**SCSQ**						
Active coping	1.80	0.61	2.20	0.50	−20.419	<0.001
Passive coping	1.45	0.59	1.32	0.57	6.509	<0.001

*SAS, Self-rating Anxiety Scale; SDS, Self-rating Depression Scale; CD-RISC, Connor-Davidson Resilience Scale; and SCSQ, Simplified Coping Style Questionnaire.*

### Mental Health, Psychological Resilience, and Coping Styles

#### Depression

Compared with the non-depressed group, the depressed group had significantly higher SDS total scores (as expected), as well as significantly higher SAS total scores, and lower CD-RISC and SCSQ total scores (Table 1). The depressed group scored lower on all three dimensions of psychological resilience, including tenacity (*F* = -27.763, *P* < 0.001), strength (*F* = -30.820, *P* < 0.001), and optimism (*F* = -25.727, *P* < 0.001), in addition to lower total psychological resilience scores (*F* = -30.648, *P* < 0.001) as well lower scores as on the measure of active coping (*F* = -27.426, *P* < 0.001), and higher scores on the measure of passive coping (*F* = 3.806, *P* < 0.001).

After controlling for age, sex, marital status, education, and total SDS score, there were significant differences in resilience scores for tenacity (*F* = 17.897, *P* < 0.001), strength (*F* = 35.064, *P* < 0.001), optimism (*F* = 47.855, *P* < 0.001), CD-RISC total score (*F* = 11.834, *P* < 0.001), active coping style (*F* = 24.414, *P* < 0.001), and passive coping style (*F* = 2.712, *P* < 0.001) between the depressed and non-depressed groups. All comparisons remained significant following Bonferroni correction (Bonferroni corrected *P* value cutoff < 0.006).

#### Anxiety

Compared with the non-anxious group, the anxious group had significantly higher SAS total scores, as well as higher SDS total scores, lower CD-RISC total scores and lower SCSQ total scores (all *P* < 0.001; Table 2). Similar to the depressed group, the anxious group had lower scores than the non-anxious group on all three psychological resilience factors, including tenacity (*F* = -22.294, *P* < 0.001), strength (*F* = -24.534, *P* < 0.001), optimism (*F* = -21.176, *P* < 0.001), and total psychological resilience (*F* = -24.501, *P* < 0.001), as well as on the measure of active coping style (*F* = -20.419, *P* < 0.001), and higher scores on the measure of passive coping style (*F* = 6.509, *P* < 0.001; Table 2).

After controlling for age, sex, marital status, education, and SAS score, there were significant differences in tenacity scores (*F* = 11.829, *P* < 0.001), strength scores (*F* = 21.455, *P* < 0.001), optimism scores (*F* = 31.908, *P* < 0.001), CD-RISC total scores (*F* = 7.688, *P* < 0.001), active coping style scores (*F* = 13.355, *P* < 0.001), and passive coping style scores (*F* = 3.358, *P* < 0.001) between the two groups. These differences remained significant following Bonferroni correction (Bonferroni corrected *P* value cutoff < 0.006).

### Factors Affecting Depression and Anxiety

Factors associated with membership in the depressed or anxious groups were next examined using binomial conditional logistic regressions. Sex, age, education, marital status, the psychological resilience subscales tenacity, strength, optimism, SAS score, SDS score, as well as the active coping and passive coping subscale scores were entered into the regression models. As shown in Table 3, the results demonstrated that being female, strength in CD-RISC and active coping styles remained protective factors of depression. However, passive coping styles and SAS score were risk factors for depression.

**TABLE 3:** Logistic regression analyses examining factors associated with depression and anxiety.

	***B***	**SE**	**Wald**	***P***	**OR**	**95% C.l.**	
**Depression (χ *^2^* = 6.064; *P* = 0.640)**						**Lower limit**	**Upper limit**
Female sex	−0.261	0.118	4.929	0.026	0.770	0.612	0.970
Strength	−0.079	0.011	48.170	0.000	0.924	0.903	0.945
Active coping style score	−0.983	0.133	54.429	0.000	0.374	0.288	0.486
Passive coping style score	0.293	0.098	8.999	0.003	1.340	1.107	1.623
SAS score	0.175	0.008	517.244	0.000	1.191	1.173	1.209
Constant	−5.444	0.447	148.290	0.000	0.004		
Anxiety (χ*^2^* = 6.347; *P* = 0.608)							
Optimism score	−0.041	0.017	5.939	0.015	0.960	0.928	0.992
Passive coping style score	0.483	0.092	27.614	0.000	1.621	1.354	1.941
SDS score	0.134	0.005	667.068	0.000	1.144	1.132	1.155
Constant	−8.061	0.385	438.107	0.000	0.000		

The logistic regression models for membership in the anxious group included optimism, passive coping style, and SDS total score (Table 3). The psychological resilience optimism subscale were protective factors for anxiety. In contrast, passive coping styles and SDS score were risk factors for anxiety.

## Discussion

The aim of this study was to investigate potential protective and risk factors for depression and anxiety among the public during the COVID-19 outbreak in mainland China. First, we found a 41.0% prevalence of significant depressive symptoms and 37.2% prevalence of significant anxiety symptoms in this population. Second, we found that female sex, strength of psychological resilience and active coping style were protective against depression, while passive coping style and anxiety severity (as measured by SAS score) were risk factors for depression. Similarly, optimism of psychological resilience was a protective factor for anxiety while passive coping style and depression severity (as measured by SDS scores) were risk factors.

The high depression and anxiety symptom severity scores in our sample (52.89 ± 15.21 and 48.77 ± 11.45, respectively), and the high rate of participants who met criteria for significant depression or anxiety (41.0 and 37.2%, respectively), confirm the previous work suggesting high rates of psychological symptoms in the context of the pandemic (Li S. et al., 2020; Qiu et al., 2020). There are multiple reasons why psychological symptoms such as depression and anxiety might be elevated in the context of the COVID-19 pandemic. First, the ongoing focus on physical health and risk of infection might itself increase the level of depression and anxiety. Uncontrollable fears associated with the unpredictability of the behavior of the virus and the actual risk of infection could cause healthy people, or those with previous subclinical symptoms, to experience anxiety and/or depression when they would not otherwise be at risk of such problems (Torales et al., 2020). Second, the uncertainty and limitations on daily life caused by the pandemic, including, but not limited to, restricted movement, need to quarantine or self isolate, limited or absent contact with friends or loved ones, supply chain shortages, could also contribute to increased rates of psychological stress, including depression and anxiety.

We found that rates of depression and anxiety were higher for women than for men, consistent with previous findings (Xiaochuan et al., 2012). Interestingly, as has also been found previously, average psychological resilience scores were lower among women were lower than those for men. For example, Wang Cui Yan (Wang C. et al., 2020) have previously reported that women experience more significant psychological distress as well as higher levels of stress, anxiety, and depression, during the COVID-19 outbreak. Considering that women have multiple roles in society (mother, wife, and professional woman) and are also affected by physical factors, psychological factors and social factors, all of which may increase the risk of depression for women (Lifen et al., 2015). However, our logistic regression analyses suggested that it was female sex that was protective against depression. From the Supplementary Table 1, it can be seen that the scores of active coping styles of women are higher than those of men. Studies have shown that active emotional regulation can not only affect the relationship between depression level and cognitive bias, but also help patients to treat life events correctly and reduce cognitive bias through certain cognitive correction and treatment to enhance their correct coping concepts (Xue, 2020). which illustrates the importance of positive coping styles in reducing the risk of depression. This result is consistent with the fact that active coping style is a protective factor for depression.

We also found that older participants in our study (>55 years) reported less anxiety and depression than did younger participants (<18 years). This result is similar to that reported in another study (Wang Y. et al., 2020) in which anxiety rates were higher in age groups below 40 years and less in age groups above 40 years. From the Supplementary Table 1, Furthermore, the average score of each psychological resilience for older participants (>55 years) was higher than that for the younger group (<18 years). First of all, Beck’s cognitive theory holds that cognitive dysfunction, as a potential and deep cognition, often affects the maintenance and development of depression. Psychological resilience can affect cognitive bias through multiple factors, and its intermediary role in the regulation of positive emotions reaches 55.18% (Xue, 2020). The authors of this study suggested that the elderly have more life experiences, which may lead to stronger psychological adjustment abilities when compared to younger people (<18 years). Another possibility is that the elderly may have limited access to acquire a constant flow of information in real time using the internet and smartphones (Yang et al., 2020), thus reducing excessive exposure to epidemic information, and subsequently reducing stressors that may trigger depression.

Finally, individuals with higher educational achievement had lower rates of anxiety and depression, as did married individuals compared with those who were unmarried. Under the impact of information flow, people with higher education years can judge more rationally and cope with the impact of the epidemic in a more reasonable way, so the level of depression and anxiety is lower. For married people, these findings are consistent with the hypothesis that increased access to resources, increased family support and external support systems may increase one’s ability to effectively cope with the life changes and emotional instability that is often engendered by the COVID-19 outbreak (Haoyuan et al., 2019). Individuals who have good social support may also have higher levels of positive emotions and enhanced social adaptability, and be more effective in alleviating psychological pressure, thereby reducing the risk of depression (Li et al., 2017). It indicated that the marriage problem of the unmarried was worth paying attention to.

Perhaps most importantly, the findings that active coping style and, in the case of anxiety, optimism, appears to be protective against the development of psychological symptomatology, independent of demographic factors, suggests potential intervention or prevention strategies. Although psychological resilience is considered to be an inherent trait, allowing individuals to pursue internal harmony and effectively adapt to changing environments in the context of life events or stressful situations (Li and Guang-rong, 2012), characteristics such as optimism can also be nurtured in individuals who may not inherently tend toward optimism. Similarly, active rather than passive coping styles can be modeled and practiced in the context of a psychotherapeutic or similar intervention.

At least one study has provided some evidence-based recommendations for boosting mental resilience can help to successfully deal with the coronavirus pandemic. We suggest that, in addition to providing information and increasing knowledge about actual risk related to COVID-19, focusing on promoting optimism and active coping styles among the public could serve to mitigate the negative mental health effects of this pandemic. Psychologists or other professionals could be called on to provide psychological education or other online interventions, aimed at increasing resilience and coping in the face of this and other potential public health emergencies. In addition, the provision of online psychological services and hotlines could provide rapid and easy-to-access counseling or intervention services for those members of the public who experience excessive stress responses or problematic or severe psychiatric symptoms (Liu S. et al., 2020).

Although this study has several strengths, including the large sample size, the assessment of potential protective and risk factors for psychological symptomatology, it also has some limitations. First, one of the limitations of this study is that the sample of the online epidemic survey is under-represented. For example, the elderly (>80 years) and a small part of rural people have limited access to internet services and smart phones (Yang et al., 2020). Therefore, although our research involves the public in multiple regions, the elderly in the sample, a small part of rural people are not involved. Second, the questionnaire was distributed at the peak of the outbreak. The trajectory of the pandemic and knowledge of the potential impact of the coronavirus have changed substantially since then, and symptom levels may have also changed accordingly. Responses to the survey may have been affected by many factors that were specific to the timeframe in which it was administered, such as the environment, mood, and understanding of the questionnaire items at that time. However, it is unlikely that the relationships between anxiety, depression, coping style and resilience will have changed, as these are not thought to be directly related to the pandemic itself. Third, the surveys were all completed using self-report questionnaires; assessments and assignment of diagnoses by psychological professionals were not feasible, and thus the relevance of these findings may be somewhat limited. This is offset somewhat by the fact that the research surveys were all submitted anonymously and the sample size was very large, potentially increasing the validity and robustness of the responses. Finally, the study was designed to be cross-sectional rather than longitudinal in nature, and regional differences throughout mainland China were not assessed for feasibility reasons. It is possible that rates of depression and anxiety in response to the COVID-19 pandemic may differ among people in different regions, as these regions also differ with regard to the severity of the epidemic and perhaps also to the response.

We also have limitations in the study. The results of other factors are not significant, but it cannot be concluded that only these factors contribute. In the future work, we can continue to expand the sample size to observe the related factors of depression and anxiety during the epidemic period.

That said, taken together, the findings of this study do indicate that, for the public in mainland China, female, strength, optimism and active coping styles may act as protective factors against the development of depression and anxiety. It follows then, that early, active, and effective targeted psychological intervention may improve mental health and coping skills in the context of an ongoing pandemic, and perhaps also for other, future external environmental changes or traumatic events (Li Z. et al., 2020). This would include providing online psychological services and/or hotlines for those experiencing excessive stress responses or problematic symptomatology, in addition to identifying resources (such as ways of increasing psychosocial support) that may reduce stressors on an individual basis. The development of online mental health services and psychological hotlines in China and elsewhere could become an important tool in emergency intervention measures for public health emergencies such as the COVID-19 crisis (Liu S. et al., 2020).

## Data Availability Statement

The original contributions presented in the study are included in the article/Supplementary Material, further inquiries can be directed to the corresponding author/s.

## Ethics Statement

This study was approved by the Institutional Review Board of the affiliated Nanjing Brain Hospital of Nanjing Medical University. All procedures complied with the ethical standards of the latest version of the Helsinki Declaration.

## Author Contributions

XY and HY prepared the setting for online survey of the psychological scale. PZ, HM, CT, and HC collected data. HO and JL for the scale issuance and quality control. SS analyzed the data of the survey and wrote the manuscript. CM, SN, NL, XZ, and NZ contributed for suggestions on revision after review of the manuscript. All authors contributed to the article and approved the submitted version.

## Conflict of Interest

The authors declare that the research was conducted in the absence of any commercial or financial relationships that could be construed as a potential conflict of interest.
