# Supplementary material for: Psychological Resilience as a Protective Factor for Depression and Anxiety Among the Public During the Outbreak of COVID-19
Source: Front Psychol. 2021 Jan 22;11:618509. doi: 10.3389/fpsyg.2020.618509 (PMC7862326; doi:10.3389/fpsyg.2020.618509)
Supplement: Supplementary file 1 [file Table_1.DOCX]

Table S1 **Demographic difference tests of mental health, mental resilience, and coping style**

|  | | n | SDS | SAS | Tenacity | Strength | Optimism | Total CD-RISC | active coping | passive coping |
| --- | --- | --- | --- | --- | --- | --- | --- | --- | --- | --- |
| Gender | Male | 886 | 41.09*±*12.11 | 38.68*±*9.35 | 34.24*±*10.93 | 23.78*±*6.83 | 10.14*±*3.60 | 68.16*±*20.19 | 2.03*±*0.61 | 1.37*±*0.63 |
|  | Female | 2294 | 42.77*±*12.17 | 39.15*±*9.08 | 31.50*±*10.11 | 22.53*±*6.37 | 9.97*±*3.50 | 64.00*±*18.76 | 2.06*±*0.56 | 1.37*±*0.56 |
| t | |  | -3.498 | -1.290 | 6.704 | 4.851 | 1.211 | 5.487 | -1.319 | -0.118 |
| P | |  | 0.000 | 0.197 | 0.000 | 0.000 | 0.226 | 0.000 | 0.187 | 0.906 |
| Age | <18 | 439 | 57.57*±*15.25 | 49.44*±*10.48 | 29.59*±*12.54 | 20.42*±*7.93 | 8.13*±*3.96 | 58.14*±*23.13 | 1.84*±*0.71 | 1.16*±*0.66 |
| (years) | 18-55 | 2583 | 52.38*±*15.12 | 48.79*±*11.59 | 32.50*±*9.97 | 23.17*±*6.18 | 10.30*±*3.34 | 65.97*±*18.30 | 2.08*±*0.55 | 1.39*±*0.56 |
|  | ＞55 | 158 | 48.14*±*13.71 | 46.58*±*11.49 | 35.83*±*9.52 | 24.89*±*6.03 | 10.80*±*3.56 | 71.53*±*17.92 | 2.21*±*0.47 | 1.50*±*0.55 |
| χ² | |  | 158.489 | 117.898 | 268.294 | 194.080 | 200.599 | 320.269 | 179.817 | 157.580 |
| P | |  | 0.000 | 0.026 | 0.000 | 0.000 | 0.000 | 0.000 | 0.000 | 0.000 |
| Education | ≤12 | 824 | 54.79*±*15.13 | 49.34*±*10.70 | 31.04*±*12.32 | 21.54*±*7.94 | 8.67*±*4.04 | 61.24*±*23.00 | 1.90*±*0.68 | 1.17*±*0.65 |
| (years) | 12-16 | 1967 | 52.63*±*15.42 | 48.78*±*11.77 | 32.54*±*9.76 | 23.26*±*5.96 | 10.40*±*3.25 | 66.20*±*17.78 | 2.10*±*0.53 | 1.44*±*0.54 |
|  | >16 | 389 | 50.12*±*13.81 | 47.58*±*11.28 | 33.47*±*8.95 | 23.79*±*5.48 | 10.96*±*2.87 | 68.22*±*16.37 | 2.13*±*0.53 | 1.40*±*0.50 |
| χ² | |  | 138.160 | 133.933 | 196.223 | 178.186 | 245.049 | 277.336 | 170.565 | 274.739 |
| P | |  | 0.014 | 0.002 | 0.000 | 0.000 | 0.000 | 0.000 | 0.000 | 0.000 |
| Marital status | Unmarried | 1067 | 57.01*±*15.41 | 50.39*±*11.47 | 30.32*±*10.74 | 21.49*±*6.74 | 9.15*±*3.58 | 60.96*±*19.80 | 1.94*±*0.61 | 1.32*±*0.61 |
|  | Married | 1953 | 50.54*±*14.39 | 47.81*±*11.15 | 33.24*±*10.10 | 23.61*±*6.27 | 10.45*±*3.40 | 67.30*±*18.56 | 2.11*±*0.55 | 1.39*±*0.57 |
|  | Others | 160 | 53.99*±*17.56 | 49.73*±*13.42 | 33.24*±*10.36 | 23.18*±*6.62 | 10.63*±*3.64 | 67.06*±*19.53 | 2.10*±*0.59 | 1.50*±*0.51 |
| χ² | |  | 283.792 | 141.360 | 168.134 | 145.801 | 136.883 | 278.114 | 202.727 | 68.239 |
| P | |  | 0.000 | 0.000 | 0.000 | 0.000 | 0.000 | 0.000 | 0.000 | 0.029 |

Note: SAS= Self-rating Anxiety Scale; SDS= Self-rating Depression Scale; CD-RISC= Connor-Davidson Resilience Scale.
